# The postcolonial sociology of love in Gandhi’s non-violent political culture of *Satyagraha*

**DOI:** 10.3389/fsoc.2025.1520969

**Published:** 2025-09-02

**Authors:** Cristiano Gianolla

**Affiliations:** Centre for Social Studies, University of Coimbra, Coimbra, Portugal

**Keywords:** democracy, duty, intercultural dialogue, moral philosophy, political emotions, political sociology, relationality, sociology of emotions

## Abstract

*Satyagraha* is the philosophy of non-violent resistance and a critical constructivist approach—building on life experience and experimentation—to social action. It was delineated by M. K. Gandhi in the first half of the 20th century and characterized the anti-colonial struggle in India. It was also mobilized to overcome polarization between religious, social, and cultural groups in search of dialogue. Finally, *Satyagraha* included postcolonial provisions for a democratic system based on duty, empathy, and social love. Gandhi’s theory and social work combined a critique of individualism, state centralism, and representation in liberal democracy and the construction of a political culture based on social love from the community level. Gandhi projected a socio-political theory of democratization through cognitive-emotional liberation from heteronomous processes of subjectivation in favor of the self-construction of subjectivity. *Satyagraha* elaborates autonomy as individual and collective self-rule or *swaraj*. On the basis of critical autonomy, *Satyagraha* constructs interpersonal and intercultural dialogue and social governance outlined by “welfare for all” or *sarvodaya*, a duty-based approach to democratization. This article analyzes the love-centered philosophy of *Satyagraha*, exploring the relevance of its key conceptual constructs in relation to the mobilization of social and political emotions and investigating the way in which it produced a comprehensive political culture of social love. Non-violence (*ahimsa*) is the method based on the duty to achieve truth (*satya*), which prioritizes love and relational emotions over force and one-sided or principled reason. *Satyagraha* generates social dialogue, promotes liberation from the domination of possession and passion, as well as the achievement of (individual, social, and political) *swaraj*. *Ahimsa* was both the key to mobilizing the entire population from the bottom up, ensuring socio-political harmony in a prospective independence, and to building a political culture in which means and ends were prefiguratively aligned. Gandhi formulated an original, utopian, and thought-provoking political philosophy, which is subject to ambiguities and limitations, stimulating provocative reflections on empathy and social love.

## 1 Introduction

The most comprehensive academic collection of essays on social love recently published (Cataldi and Iorio, 2022a) concludes by asserting that love overcomes inter-group partiality, induces the “social ecology of caring” as a relational principle, focuses on suffering from the last and excluded, and translates into actions to fight inequality and generate diversity and utopia (Iorio and C., 2022). The collection also points out that social love is not a sociological normative idea, but that it is a categorization emerging from the sociological imagination in order to frame social reality from empirical observation. This means that the use of (social) love as a term is not the sole indicator of its use as a sociological category. As a social activist who was able to instigate an articulated socio-political culture at a time when sociology was being founded as a discipline, Mohandas Karamchand Gandhi (1869–1948) profoundly contributed to the profile of social love outlined above. The scope of the present article is to unveil Gandhi’s postcolonial contribution to the political sociology of social love. It is, in fact, surprising that the book edited by Cataldi and Iorio cites only an epigraph by Gandhi in the last pages, while his contribution has no real place in the volume.

Gandhi the “Mahatma” (great soul, a title assigned by the multitude and officialized by Tagore to outline his spiritual and moral qualities) is often cited as a reference on social love, while his thought is not duly analyzed. Another example is the relevant work by Adrian Scribano (2020) *Love and Collective Action*, in which, again, Gandhi appears only through an epigraph. While Cataldi and Iorio focus on *agape* and Scribano on *philia*, they analyze similar social phenomena. These and other theorists of love in society identify social love as a critical and collective energy that exceeds social expectations as it overcomes the principle of reciprocity. It is therefore intolerable for the calculated, hegemonic, and normalized logic of power and subjectivation controlling the dominant regime of truth. Social love is an epistemic action, produced by performative and interstitial practices of resistance to oppression generated by political power. Social love constructs a social vision and demands social change based on inter-group diversity, mutual recognition, and relationships between individuals and social groups (King, 2004). Gandhi’s social love is a good example of how emotions of openness toward Otherness—compassion, empathy, and recognition of difference—encourage societies to embrace diversity while challenging systems of exclusion and inequality.

Contrary to these more recent omissions, Gandhi was a reference in the seminal work by the founder of Sociology at Harvard University, Pitirim A. Sorokin, who—as Gandhi—was influenced by Leo Tolstoy. In *The Ways and Power of Love* (Sorokin, 2002), Gandhi’s social love perfectly aligned with the entanglement of subjective and objective ends, which Sorokin typified as intense, extensive, enduring, unselfish, and wise. As an anti-colonial activist and thinker, and a seminal reference for postcolonial scholarship, Gandhi elaborated on social love outlining processes of subjectivation, value for diversity, and a break with the binary inter-group opposition logic, all elements that characterize the recent study of social love (Cataldi, 2022, p. 28–29). The sociological study of love is exposed to polysemic interpretations and is a belated topic of Western sociology, which is still at the early stages of its research in this field (Rusu, 2018). The neglect of Gandhi and the marginalization of Sorokin seem to posit a Western-centric limitation to sociology. Gandhi showed that social love is not an unknown phenomenon in non-Western social and political thinkers; he made a case for mobilizing love against colonial oppression. Moreover, love has been approached as a dimension that pertains to the private life sphere—be it family or religious belief—separate from the public one. It must be noted that Gandhi was also criticized for failures as a family person, his stubborn personality, his discriminatory attitude toward the natives of South Africa in his early years, his vision on caste, and his sexual abstention experiment toward the end of his life (Adams, 2010; Desai and Vahed, 2016; Kumar, 2006; Roy, 2014). Politically, Gandhi has been historically criticized by the left and the right, until now (Guha, 2023). While these debates are ongoing, rebuttals show that, for example, Gandhi became a reference for the African National Congress (Nauriya, 2016; Nauriya, 2015; Nauriya, 2012; Kolge, 2016a, 2016b; Kolge, 2014). Moreover he was esteemed by the Afro-American sociologist Du Bois (1957, see also Lal, 2021) as well as the African American anti-colonial and anti-racist struggle (Lal, 2021), including in Latin America (Bissio, 2021), and he is still regarded as a leading figure for India’s future (Khare, 2021; Rai and Tiwary, 2021; Patkar, 2021).

Gandhi was educated in India and England and worked as a lawyer in South Africa for 20 years before returning to India in 1914 to support and steer India’s anti-colonial struggle. His sources included Western and non-Western authors and texts: religious books (such as the Bible, Upanishads, and Gita), religious personalities (such as the Buddha, Jesus, and Muhammad, the prophet of Islam), and ancient and modern political and spiritual thinkers like Edward Carpenter, Gopal Krishna Gokhale, Giuseppe Mazzini, Plato, Raychandbhai (or Shrimad Rajchandra), John Ruskin, Henry Stephens Salt, William Mackintire Salter, Robert Harborough Sherard, Henry Thoreau, Leo Tolstoy, and others (Gandhi, 1938, Appendix I; 1927, Chap. 20, 26, 40, 47, 82, 95). This is the background of the intercultural and interreligious thinking that led Gandhi to inductively theorize *Satyagraha* as a philosophy of social struggle and democratic organization based on social love. In his seminal and anti-colonial book *Hind Swaraj* (Gandhi, 1938, Chapter XVI), Gandhi described *Satyagraha* as “love-force, soul-force, or, more popularly but less accurately, passive resistance.” Years later, as he described how the very notion of *Satyagraha* emerged, he affirmed, “Truth (*Satya*) implies love, and firmness (*agraha*) engenders and therefore serves as a synonym for force” (Gandhi, 1950, p. 106).

During the late 19th and early 20th centuries, India was undergoing intense socio-political upheaval under British colonial rule, marked by economic exploitation, social fragmentation, and rising communal tensions. Gandhi emerged in this context as the leader of a moral and political movement, advocating for *swaraj* (self-rule) not just as political independence but as a deeper transformation of the Indian self and society (Dhareshwar, 2010; Nigam, 2009). Gandhi’s movement was driven by social love combined with a critical assessment of, and opposition to, colonialism. He also held a critical view of liberal representative democracy (Pantham, 1983; Rai and Tiwary, 2021), characterized by a non-violent state (Srivastava, 1968) and a bottom-up conceptualization of democracy as opposed to top-down (Roy, 1984; Parekh, 1989; Skaria, 2016). Gandhi was contextually involved in socio-political and historical struggles, and his writing was strictly connected with his social action (Chadda, 2010). Gandhi’s emphasis on inter-religious harmony, his critique of modernity, and his village-centric economic vision were all responses to both colonial domination and the internal hierarchies of caste and class (Chakrabarty, 2006; Biswas, 2018). They were intended to create a political culture of *Satyagraha*. Nathuram Godse, a member of the Hindu nationalist movement *Rashtriya Swayamsevak Sangh* (RSS) defending *Hindutva* or Hinduness, assassinated Gandhi because he promoted a political culture of social love and inter-religious dialogue. The RSS was banned for a few years, and its leader could re-establish the legitimacy of the movement only by co-opting and appropriating Gandhi’s legacy, although much in contradiction with Gandhi’s views (Chaudhary and Narayan, 2024; Parel, 2003). Therefore, Hindu nationalism after Gandhi has been characterized by an ideology that repudiates Gandhi’s non-violent and communal harmony principles and examples (Sen and Wagner, 2009). In fact, Gandhi is a hate figure of current Hindutva politics promoted by the RSS and the nationalist, governing (since 2014) Bharatiya Janata Party (BJP) led by Narendra Modi (Ayyub, 2024; Guha, 2023; Singh, 2022). In Gandhi’s view, freedom from colonial rule was only one part of the full independence process (Gandhi, 1938, 1994). Gandhi aimed to generate a political culture to deconstruct the hegemony of modern Western political culture and strengthen self-consciousness of the Indian people (Hardiman, 2003, p. 71; Parekh, 1989, p. 208–209). Independence included emancipation from colonial ideas, epistemic decolonization, and the development of a democratic vision centered on self-rule through a duty-based approach. This political culture is compatible with a morally oriented political representation, but far removed from a strong representative, procedural, and elitist model of democracy.

Gandhi influenced the political trajectory and culture of India in several ways, including the spirit of the constitution, deliberative democratic practices, social and political practice (reducing sectarianism and exclusion), and isocio-cultural dynamism and the spirit of decentralization that led to the devolutionary and participatory *panchayati raj* (Gupta, 2013, p. 45–67; Mehta, 2022; Prasad, 2011; Rudolph and Rudolph, 2006, p. 20–31). A sociological appreciation of Gandhi’s work highlights his fundamental legacy in India’s liberal and democratic institutions (Gupta, 2009). Nonetheless, the non-violent love-based political culture he desired to establish was evaded by country leaders after independence. His political philosophy was visionary of the mechanical and dehumanizing trajectory of modern politics; however, his alternative proposal remains in the realm of utopia (Runciman, 2018).

A previous analysis focused on the intercultural dimension of Gandhi’s discourse, distinguishing it from demagogic populism and explaining why it is an alternative (Gianolla, 2020). The present article builds on that and other work in order to analyze Gandhi’s instigated political culture of *Satyagraha* as social love, characterized by “non-violence and no hatred” (Rai and Tiwary, 2021, 123). This concept, which has generally been used to define the non-violent philosophy and social action of Gandhi, is, in fact, very insightful for analyzing how Gandhi constructed a political culture of social love and empathy through duty. It has also been very impactful in politics and social action worldwide, as a recent Special Issue demonstrates (Mohanty, 2021). The following sections, respectively, explore the following topics: (a) elaborate on the well-known—however deeply relevant—relationship between truth and non-violence in Gandhi’s thinking; (b) analyze in detail the sociological construct of *Satyagraha*, with a special focus on the sociology of emotions; and (c) investigate the ontological origin of social love in Gandhi by focusing on the centrality of relationality in his theory. The final discussion critically assesses the previous arguments in relation to the liberal debates on rights and freedom, and the article concludes with remarks on the impact of Gandhi’s *Satyagraha* on democratic theory.

## 2 Truth and non-violence in democratization processes

Two concepts sustain Gandhi’s philosophy and socio-political action: “*sat*” or truth and “*ahimsa*” or non-violence. For Gandhi, “Truth is God,” and therefore he believed that the superior goal of life was only achievable through the superior means of non-violence. In concluding his autobiography, Gandhi affirmed: “I can say with assurance, as a result of all my experiments, that a perfect vision of Truth can only follow a complete realization of Ahimsa” (Gandhi, 1927, Chap. 168). This work demonstrates the empirically informed character of Gandhi’s political philosophy that encloses politics, spirituality, and morality in the two key elements: truth—the aim—and non-violence—the means—indestructibly united in political terms. However, the method—non-violence—was the primary political objective because it is the gateway to truth and is produced in political practice. The experimentation, practice, and dissemination of *ahimsa* constituted the very prefigurative construction of Gandhi’s political culture of social love.

*Ahimsā, indeed, was the concept—both ethical and epistemological because it was defined within a moral and epistemic practice that was wholly “experimental”—which supplied Gandhism with ·a theory of politics, enabling it to become the ideology of a national political movement. It was the organizing principle for a “science” of politics—a science wholly different from all current conceptions of politics which had only succeeded in producing the “sciences of violence,” but a science nevertheless—the “science of non…violence,” the “science of love”* (Chatterjee, 1993a, p. 107).

Gandhi’s “science of love” was aimed at generating *sarvodaya*, service, duty, and welfare for all in the socio-political organization. Skaria (2002) has termed “neighborliness” the socio-political practice that Gandhi elaborated to carve this political culture within diversity and to deal with power. In fact, Gandhi’s approach exceeded the solidarity for the (political) in-group that Honneth (1995) frames in the theory of recognition. It is also different from what Honneth identifies as the “solidary with suffering people somewhere else,” termed as “sympathy or even *Nächstenliebe*,” in the Christian sense of “love thy neighbor” (Iorio and Campello, 2013, p. 250). Gandhi’s radical project of social equality (Skaria, 2016) is absent in Honneth’s theory of recognition. Gandhi aimed to upscale intra-group solidarity to achieve inter-group love based on *tapasya*, the renunciation of violence through self-sacrificing and suffering. “The *tapasya* of neighborliness differed depending on the kind of absolute difference and power relation being addressed: the equal was met with *mitrata* (’friendship’), the subordinate with *seva* (’service’), and the superior with *Satyagraha* (’civil disobedience’)” (Skaria, 2002, p. 957). While agreeing with this typology, this article argues that *Satyagraha* can be understood within the overall framework elaborated by Gandhi to shape how subjective agency builds critical postcolonial relationality and interculturality.

Gandhi’s instigated political culture of social love merged Western humanism and Hindu worldviews in opposition to Western colonialism, Muslim separatism, and high Hindu casteism (Parel, 2003). *Ahimsa* is an active force of love, the renunciation of violence and its root causes: excess of possession and passion. As underlined by Bhikhu Parekh, “detachment it did not mean indifference but absence of attachment, not lack of interest but of self-interest” (Parekh, 1989, p. 97), and his thinking is part of a cosmic order searching for harmony with fellow humans, all living beings, and Nature (Shiva, 2021). The economic view is grounded in dialogical ontology in which relationality characterizes the moral, cognitive, and emotional structure of society. Relationality not only characterizes the relation between subjects but also between subject and the social, material, and symbolic worlds. Parel argues that Gandhi achieved this through the philosophy of *purusharthas*, or life purposes. Gandhi sought to balance *dharma* (ethics), *artha* (wealth), *kama* (passion), and *moksha* (spiritual liberation). For Gandhi, Western modernity has lost moral and spiritual value, and he advocated a political culture reconciling these two dimensions with prosperity and materiality (Parel, 2003). Likewise, Indian civilization has degenerated due to the downplay of *kama* and especially *artha* (Parel, 2003); thus, Gandhi advocated a social theory of equilibrium between the purposes of life. He instituted this approach in his *Ashrams*, through social and political action, and was followed by a growing number of direct collaborators and emulated by countless fellows in India during the freedom struggle. He instigated the political culture of *Satyagraha* through this work.

The prominence of the spiritual dimension in Gandhi induces religious pluralism and “epistemic humility.” “Gandhian humility negates claims to absolute power and emphasizes the need for dialogue with others” (Jahanbegloo, 2018, p. 99; see also Bilgrami, 2009). Gandhi also made a connection between body and mind, reason and emotion, in a religious pursuit through common sense. In a speech about the interreligious dialogue between Muslims and Hindus, he affirmed:

*My innermost urge is for pure non-violence. My weakness is that I do not know how to make it work. I use my intellect to overcome that weakness. If this intellectual cleverness loses the support of truth, it will blur my vision of non-violence, for is not non-violence the same as truth? Mere practical sense is but a covering for truth* (Speech at Gandhi Seva Sangh Meeting 28 March 1938—CWMG Vol. 66, p. 445).

In this passage, Gandhi equated non-violence and truth, indicating that the “practical sense” is superior to intellectual evidence. Moreover, he used the word “urge” to identify his attachment to non-violence; this suggests that there is an emotional attachment in the individual connection with the transcendental moral principles. It is a bodily—urge as a strong desire or impulse, rather than purely ascetic affect—validation of moral principles which must be reflected in spiritual practices. The political implication of this is that *Satyagraha* is not simply political dissent, but “Gandhi viewed *Satyagraha* essentially as an ethical commitment and a constructive political action. For Gandhi, the spiritual and the political were the same” (Jahanbegloo, 2018, p. 96). In *Satyagraha*, reason and emotions are both conducive to truth, and when the former fails, the latter holds a practice-based capacity to shed light on the moral course of events. The political dimension is ingrained—as opposed to separate—in the moral one; it is a consequence of it.

The emancipation of the individual and their subsequent availability for providing social contributions is of central importance. A new educational model must therefore achieve training through which practical work is valued and frees the subject from any source of servitude (domination from outside and artificial needs) and which educates for “real life” (H 10-3-1946 and H 2-2-1947). In “*nai talim*” (or *nayee talim*) —Gandhi’s education scheme—practical education combined with literary and spiritual education aimed to tutor one as an independent and freely cooperative member of the democratic polity. This was the model he practiced in his *Ashrams*, characterized by duty. The new educational model started at the local level, in the community where there were the conditions for “abolishing the eternal conflict between capital and labor” (Gandhi, 1945, p. 20), where possession and accumulation could cease to be an objective and become a mere instrument of subsistence. The purpose of this educational system for Gandhi was “university education [whose aim] should be to turn out true servants of the people who will live and die for the country’s freedom” (H 25-8-1946). Partha Chatterjee maintains that modern education for Gandhi “both exaggerated and rationalized the inequalities in society” because “[it] ignores completely the ethical aspect of education and the need to integrate the individual within the collectively shared moral values of the community” (Chatterjee, 1993b, p. 92). As a result, this model of education produces self-interested individuals and their disaffection from society, as well as moral anarchy. In Gandhi’s view, intellectual education should be equally available to all people and lead to human self-fulfillment, not to greed and possession. Gandhi’s view on education is thus bridging mind and body, reason and emotion; both have to be educated as opposed to separating the education of the mind—reason for the pursuit of specialized knowledge—which results in segregated societies.

## 3 The sociology of emotions of *Satyagraha*

*Satyagraha* is a concept and method coined by Gandhi. It is the method of practicing non-violence in politics. It is formed by two words: *satya* (truth) and *agraha* (firmness, desire, insistence, or determined pursuit), resulting in “firmness on truth,” “desire of truth,” “insistence on truth,” or “determined pursuit of truth”. While “Truth and nonviolence are as old as the hills” (H 28-2-1936), *Satyagraha* points to the end (*satya* or truth) through focusing on an emotionally charged motivation (firmness/desire/instance/determined pursuit of truth). Despite the austerity of Gandhi’s philosophy in relation to desire and possession, *Satyagraha* shows how desire is central to Gandhi’s philosophy. The control of emotions related to materiality (passion and possession) is outweighed by the centrality of emotions related to spirituality and moral pursuit. Emotions are the mediators of the *purusharthas*, and they are not dismissed but reframed in relation to the role they play in dominant Western political cultures or decaying Hindu civilization. Gandhi provided an original normative understanding of political emotions; he identified how the habituated use of emotions leads to power domination in politics and proposed an approach that allows emotions to disrupt political power. This approach protects the relations between people over individual materiality, rejecting violence as the last resort for self-protection. It defuses the conditions for the brutal clash of relations generated through violence. Gandhi’s *Satyagraha* deconstructs fear—subverting its potential to generate political passivity and the domination of violence—in order to build on fearlessness. Gandhi provided an approach that radically deconstructs the foundations of political emotions upon which political power reproduces itself, including within liberal democracy. For example, Gandhi dismissed the ontological insecurity generated by exclusionary populist discourse through a constructivist ontological security of non-violent self-rule rooted in spiritual and moral principles. Gandhi’s philosophy fosters emotions through agency, subjectivity, and relationality; he proposes trust, empathy, forgiveness, compassion, and love through fearlessness. Political responsibility—a “surrender without subordination” as Skaria (2014, 2016) defines *Satyagraha*—is, in fact, what can also be identified as democratic duty—and is the building block of the non-violent and intercultural society.

The emotional strategy of *Satyagraha* is inherent in the reasons that led Gandhi to search for an alternative to the term “passive resistance” to describe the growing experience of self and social articulation that backed the civil resistance of the Indians in South Africa. In the first place, it was at times translated with the resistance of the “weak,” which is misleading. Secondly, passive resistance could still envisage recourse to violence as a means of struggle, which was unacceptable to Gandhi (see also Suhrud, 2005, p. 310–312). *Satyagraha* is not the renunciation of violence because of personal or societal weakness, but the adoption of non-violence as a courageous action to achieve the superior end (truth). *Satyagraha* condenses both truth and non-violence; it states an attitude for the first but is used as a Gandhian synonym for the second. Both theory and practice of *Satyagraha* emerged in South Africa. Gandhi first wrote about it in the form of an article when he was incarcerated in the Yerwada prison and then published it as *Satyagraha in South Africa* (Gandhi, 1950). In 1906, Gandhi announced a contest to identify an appropriate word to better denote the non-violent struggle he was leading with Indian communities. Maganlal Gandhi won the competition with *sadagraha*, meaning “firmness in a good cause” (Gandhi, 1927, Chap. 103), which he then finalized as *Satyagraha* for the transcendental end rather than a finite one; he substituted the good cause with “truth.”

As a method of civil disobedience, *Satyagraha* is a struggle without the will to harm. It is aimed at converting the heart of the adversary through self-suffering. *Satyagraha* underlies the recognition of the inner power of the subject confronting oppression and injustice. Realizing this power, *Satyagraha* opposes resistance, without fear or passiveness, until the oppression can no longer endure. *Satyagraha* is ontologically relational; it is based on the preservation of the relation with the other regardless of the attempts that the other makes to break it through violence. However, *Satyagraha* is also relationally emancipatory; it implies the recognition that power is shared between oppressor and oppressed. Unless both parties assume that power stands with one party only, the oppressor, *Satyagraha* recognizes and rejects “voluntary servitude” (Boétie, 2011). The oppressed wins the emotional oppression (fear) and claims equality with the oppressor; this induces the potential to emancipate both from the oppressive power relation—which affects the oppressor as well. *Satyagraha* is a fundamentally democratic reassessment of power distribution. *Satyagraha* does not justify the use of violence to achieve a just or ethical demand, it preserves the relation together with the objective. Violence breaks the relation; thus, it is an impossible means to achieve truth (or emancipation or justice)—which itself is relational. Basic conditions for the practitioner, the *satyagrahi*, include: no hatred for the opponent, struggle for a true and substantial issue, resistance against offensive and violent acts without reacting, and accepting the consequences of infringing a law. A *satyagrahi* continues to be open to dialogue and compromise on honorable terms, is very critical of themselves and their own mistakes, and is very open and gentle toward the mistakes of the opponent. The struggle of the *satyagrahi* may take a very long time, but Gandhi maintained that if held with courage, there is no possible defeat for *Satyagraha* (H 18-8-1946). This is due to the primacy of the means together with the primacy of the aims, preserving non-violent means while protecting and prioritizing the relation; this is a success in itself; no defeat is possible. As quoted above, Gandhi focused on realizing “truth” in human “relationships” through non-violence: “we have no means of realizing truth in human relationships except through the practice of ahimsa” (H 23-6-1946). Truth exists in relations, not beyond them, and violence moves beyond relations, thereby concealing Truth altogether. Historical examples of campaigns are the Champaran *Satyagraha* (1917) against the exploitation of indigo farmers in Bihar, the non-cooperation movement (1920–1922), during which Indians did not cooperate with British colonizers, and the Salt *Satyagraha* (1930) against the British monopoly on salt collection in India.

Gandhi made the experimental and experiential practice of the methodology of *Satyagraha*. He created “constructive” communities where the social and political practices were experimented with before being reproduced on different scales around the country, impacting the political culture. He worked on morality by reconstituting common sense to induce a self-generated personal conscience and self-regulated subjectivation processes. Bilgrami (2002) convincingly elaborates on the centrality of example as opposed to principle in Gandhi’s moral philosophy. For Gandhi, the moral example replaces the moral principle as understood in the tradition of the Kantian imperative. The moral principles are universal and imposable on others who can be critically judged accordingly. On the contrary, the moral example is acceptable to the conscience of others through a self-generated effort. In this way, the “[e]xemplary action takes the place of principles. If someone fails to follow your example, you may be disappointed but you would no longer have the conceptual basis to see them as transgressive and wrong and subject to criticism” (Bilgrami, 2002, p. 88). This reasoning leads to considering truth as moral and experiential rather than a cognitive object linked to a universal principle. This points to the constructivist approach of Gandhi to truth and the practice of non-violence, shedding light on how it is disjoined from ideological, fanatic, and conservative religious belief.

The role of experience is visible in the proximity; this is why Gandhi’s political culture advocates the small case and its construction built on the *ashram*. The *ashram* was both a concept and a place. As a concept, it was the community model to develop a practice-based and value-oriented non-violent training. As a place, it was the community-based collective in which Gandhi lived, experimented, experienced, and worked. With the *ashram,* Gandhi questioned the concept of the state, the duality with civil society, and the rigid division of the public and private spheres. *Ashrams* were places for high-intensity political practices, characterized by the intensity level of the democratic political culture that they instilled. *Ashram* life was regulated by a moral code leading to self-restraint from worldly possessions and passions. *Ashrams* were the laboratories of a new decentralized polity for India. *Ashramites* had to respect a number of vows that regulated individual moral conduct (Gandhi, 1932; Gandhi and Desai, 1959); they touched upon truth, nonviolence, *Brahmacharya* (sexual abstinence and control of all senses in Gandhi’s acceptance), non-stealing (broadly implying restraint from material desire), non-possession (poverty and self-restraint), bread labor (social service and working for a living, non-accumulation), control of the palate (separated from other senses because the most complicated to control), courage or fearlessness, inter-religious equality, *swadeshi* (use of local products and care for the community), and removal of untouchability (instigating inter-caste living). Gandhi convened individuals and, most importantly, families of different castes, religions, languages, and regions of the country. The *ashram* provided an equal space for men and women in order to reinforce the role of women in Indian society and public life. They were also places of inter-religious and inter-caste living contributing to the struggle against communalism and caste oppression. *Ashrams* were laboratories of commitment to independence, dispute resolution, and religious, economic, social, and political development. “Gandhi came to believe that *ashrams* patterned on his ideals should primarily serve a pedagogical function by tackling the problems of the villagers, showing them the way to develop self-confidence and thus self-reliance, and training children and adults alike to be community workers without alienating them from their own people” (Thomson, 1993, p. 296).

## 4 Relational ontology and constructive subjectivation

Democracy for Gandhi is power spread horizontally and operationalized through relationality as a methodological politics of equality. Relationality is thus the grammar of social love; it institutes democracy and characterizes the entanglement between self and other. “Politics is based on the internalization of the inclusion of the other in the political project which finds expression in and is supported by the self-institution of democracy” (Jahanbegloo, 2015, p. 65). Rejection of violence emerges from dialogical ontology and produces a dominant ethical-political perspective with respect to the state’s monopoly of violence. It implies a paradigm shift, the renunciation of violence, while protecting the relation with the other to achieve one’s own goal or right. This is linked to the domination of desire. The control of violence is not done through the use of violence but through its prevention. Not in a hierarchical form—assuming the social contract as a response to the state of nature—but generative—assuming that democracy provides a horizontality capable of constituting a concept and practice of justice in which internal control—given by the non-violent method—reduces or eliminates the need for external control and makes the individualistic contract reductive.

Skaria analyses *Hind Swaraj* to understand Gandhi’s criticism of modernity from a progressive perspective on the *swa* or proper:

it breaks with the modern tradition of conceptualizing domination as the taking away of power and agency, and of conceptualizing resistance as the recovery of agency. Instead, it questions domination by insisting on a subaltern responsibility for subordination. Here, subordination is thought not as the loss of power but as the loss of the *swa*. A politics of resistance, such as that involved in *Satyagraha*, attempts to redress this loss by staying in a constitutive separation, and by giving this separation also to the dominant (Skaria, 2007, p. 222).

The root of protest and political opposition is therefore centered on self-conversion as opposed to the conversion of the opponent, which should follow. It is however questionable the extent to which this may be valid in specific inhuman relations, such as those produced by Nazism (Mukherji, 2022).

Gandhi’s *Satyagraha* favors a pragmatic outcome, as it is based on respect for the dignity of all parties in the struggle and also on the dignity of their relations during and after the struggle. *Satyagraha* re-enacts relations that oppression has impaired. Winning the opponent does not entail their destruction but the reconstitution of relations with them. This implies a “conversion” toward truth, not a situated partiality, but the reaffirmation of the dignity of the parties that is superior to the partial interests of the struggling fronts and therefore intelligible and reachable by both. In human interaction Truth becomes possible if relations are shielded from violence.

Gandhi constructed a non-violent mass movement. Non-violence was both the key to mobilize the population (as opposed to leaving the political initiative only to the elite), grant political stability in a perspective independence and build a political culture autonomous from the one of the colonizers (Bilgrami, 2002, p. 81). He adopted a rational concept of the human being who orientates their action toward a moral concept of truth (Bilgrami, 2002). While for Gandhi “Truth is God,” the rationality inscribed in his democratic vision is anthropological, as a being who *moves*—*voluntarily*—*toward* truth, and who is not *moved by truth*. Autonomy must be understood here in contrast with heteronomy. In Gandhi as for Castoriadis (1987), political action develops within society as opposed to any extra social principle, regardless of the capacity of society to acknowledge that. Autonomy grounds relational agency, because from the development of individual self-reflective subjectivity, and though personal agency, emerges the autonomous (as opposed to heteronomous) social order. “Therefore, both Gandhi and Castoriadis do not accept to call ‘democracy’ a social and political system which is imposed from outside by forcible methods” (Jahanbegloo, 2018, p. 52). This encourages criticism of liberal individualism and privatization—and capitalism—responsible for propagating social and political passivity.

*Satyagraha* is a process of self-transformation through the spiritual, social and political engagement of the “person”—as opposed to the “individual”—building their non-subjectifiable but emancipated (self-rule based) political relation with the state (Roy, 1984, Chap. 6). *Satyagraha* is the method through which social deconstruction and reconstruction follow incessantly. Dismissal of violence is instrumental for the recreation of relations as a socio-political gateway to Truth. *Satyagraha* emerged through Gandhi’s elaboration of Western and Eastern principles and therefore is culturally translated everywhere these principles are upheld, where there is a balance of spiritual, ethical, material (wellbeing) and affective purposes of life. Gandhi developed the constructive program (Gandhi, 1945) to use *Satyagraha*’s force to spread this equilibrium in the political culture of Independent India. He aimed to foster the democratization and development of Indian society, especially the rural villages which were the underdogs of the social structure. The constructive program included 18 points that were meant to ensure sustainability and equality, strengthen social bonds for community unity, and uplift Dalits and women. The program also touched upon moral, economic, sanitary and educational objectives.

Politics within institutions and politics outside of institutions, for Gandhi, were both characterized by the philosophy of *Satyagraha* and were aimed at the achievement of *swaraj*—or self-government—democracy. Constructive work was the basis for the reconstruction of Indian society confronted with the epistemological—beyond socio-political—consequences of colonialism. Gandhi’s non-violence was articulated to go beyond liberal constitutionalism and struggle against the liberal pillar of the representative colonial regime together with its “cognitive enslavement,” reaching an extra-constitutional (therefore more democratic) achievement (Bilgrami, 2002, p. 80). Gandhi clearly indicated that *Satyagraha* through the constructive program should follow after independence. For example, 3 months after India’s independence, on 13 November 1947, he affirmed, “[w]e had recognized the need for constructive work when we were slaves. We will need it many times more to transform swaraj into *surajya* [good governance/state]” (CWMG Vol. 90 p. 24).

Gandhi’s philosophy is echoed in the relational and postcolonial philosophy of the 20th century. The postcolonial ethics of liberation (Dussel, 1996, 2013) redefines the collective political subjectivity from the alterity of those excluded from modern Eurocentric political philosophy. Enrique Dussel draws on the ontology of alterity—the relational philosophy of Levinas (1969). The subject is rethought from exclusion and this recasts the ontology on which political practice and principles are also based. The subject is constituted by its ontological relation with the other, which implies a bifurcation. If the relation is accepted, it permits positive recognition of the duty toward alterity (the democratic principle is made possible). If it is rejected, it entails the negation of duty toward the other, generating the struggle against exclusion and oppression in response. Negative duty is therefore connected with an ontological rights-claim (Arendt, 1973) which entails the recognition of the relational ontology. In Levinas’ infinity (1969) and Dussel (2013) ethic of liberation, victims of the world’s systems struggle for democratic norms and institutions that are inclusive and without “externalizations,” based on a relational ontology of alterity. Being exists in relation to otherness and this shakes philosophical principles as general principles that can only be realized in this relation. Thus the relevance of experience in Gandhi and hence Dussel redefines political action in the “critical consensus of the excluded” (Dussel, 2007). This brings the moment of deconstruction and the redefinition of the political from the negative duty, that is, the duty linked to the deconstruction of the order of law, to positive duty, in view of the reconstruction of the democratic principle. The result is characterized by participation and inclusion in the political sphere and extension (as opposed to limitation) of the political sphere based not merely on (human) rights but primarily on duty (Selbourne, 2001). Being a collective and participatory effort, to take political horizontality seriously, the idea of duty is central to thought processes of democratization where elitism and verticality are displaced. Horizontality and duty are the constitutive pillars of the deeply democratic non-violent political culture of *Satyagraha*. The measurement of democracy is the operational level of these principles; the excluded of Dussel are the Dalits for Gandhi, as are Muslim people.

Also Judith Butler’s work on the ontology of social relations defends interdependence and relationality against individualism. Challenging individualism, Butler disputes self-sufficiency and proposes mutual dependencies as the constituting ethical ground of equality and non-violence (Butler, 2020). As argued above, in Gandhi’s political philosophy, while violence destroys relations, non-violence focuses on experiential truth which ultimately consolidates relations between the parts. For both Butler and Gandhi, non-violence is a constitutive force of the social relational (or dialogical) ontology because violence constitutes isolation and leads to self-destruction. Violence is the destructive force of the social by breaking relationality through individual self-defense and self-interest. The opposite force is creativity—or social imaginary significations as outlined by Castoriadis (1987, p. 203)—which need to be explored by experiencing relationality from autonomous, self-regulated subjectivities.

## 5 Beyond liberal debates on rights and freedom

Gandhi’s *Satyagraha* articulates an open agenda for political action marked by social love, which has been historically operationalized and remains available for socio-political postcolonial and post-exclusionary practice worldwide. Santos (2022, p. 204) maintains that “Gandhi symbolizes the most radical rejection of the imperial North in the twentieth century” and he invites us to think beyond political categories caged in a precise historiographic Western perspective, be it liberal or socialist. Gandhi claimed for India a proper historiography and a political categorization consistent with it. He wanted to show that the political hegemony developed in the West through colonialism was questionable and deleterious for the West as well as for India. Santos also recognizes that Gandhi forged a path that distanced the epistemological perspective from the Western-centric political categories by unlearning the colonial political categories and re-learning from the spirit of humanity that is as “old as the hills.” While debates about the legacy of the colonial and slavery past are advancing at different paces in Western countries, a Gandhi-inspired duty-based approach to democracy allows us to envisage a political and epistemological change in which diversity is protected as a political end.

A superficial reading can dismiss the relevance of *Satyagraha*’s philosophy as idealistic. However, the philosopher Dallmayr (2022) is among those who allow us to further appreciate Gandhi’s theory. He has analysed the relation between Gandhi’s political philosophy and liberal democratic thought, inscribing Gandhi’s criticism of liberal democracy within a philosophical debate in which he includes John Dewey, Hannah Arendt, Charles Taylor, Michael Sandel, among others. Dallmayr points out a range of limitations of Western political thought that invite what Sandel calls the “voluntarist conception of freedom” (Sandel, 2006) and Dallmayr reconducts to “*laissez-faire* ideology” advocating civic virtues, however disjoined from social love. This ideology limits human freedom and political agency by addressing only external limitations to freedom and neglecting those internal to the subject. Arendt (1998) has analyzed the entanglement of freedom and agency, focusing on human interaction. Outlining the role of relationality, she has demonstrated how freedom emerges in relation to others, thus criticizing the “atrophy of active political life and the truncation of freedom through its confinement to private self-seeking and self-interest” (Dallmayr, 2022, p. 143). But relationality should not lead to overlooking agency from within, and Taylor (1991) criticism of Berlin’s distinction between positive and negative liberty (Berlin, 2002), argues Dallmayr, is conducive to understanding that obstacles to (negative) freedom may also arise from within the self; these include emotions, inclinations, and habitus. Freedom is thus reconciled with a dialogical ontology that overcomes the limits of self-centered, atomized, and substantivist-individualist ontology. Dewey (1976) is among the theorists who contributed the most to understanding democracy as an ongoing process of subjective, relational, and social growth that would come closer to *Satyagraha*’s political culture. Reconnecting Dewey with Gandhi, Dallmayr highlights the substance of agency in the creation of democratic habit:

*democratic self-rule has to involve a practice of self-restraint and self-transformation (even self-emptying) capable of instilling the habit of nonviolence (ahimsa) and generous openness toward others. […] For Dewey, as we know, such a disposition or civic habit is not a ready-made “natural” endowment but a human potentiality requiring continuous struggle and lifelong educational cultivation. Treated as such a potentiality, self-rule or swaraj opens up the horizon of a possible future democracy* (Dallmayr, 2022, p. 145).

Dallmayr’s analysis indicates the genius of Gandhi’s non-violent political culture, which this article frames within the category of *Satyagraha* as the articulation of duty, empathy, and social love. Gandhi reconceptualizes subjectivity in order to overcome the self-referral individual interested in their own needs and desires through hetero-directed subjectivation processes. A subject unlearned or unhabituated to reconcile the externalities of their ontology (relationality and post-anthropocentrism) with the internal potential to contribute (agency related to motivations and emotions) to the realization of it.

## 6 Conclusion: toward a love-centered democratization

At the end of the first quarter of the 21st century, Gandhi’s *Satyagraha* may be mistakenly considered within the range of irrationality. In fact, *Satyagraha* has forged historical struggles of Black people in South Africa and in the USA (Du Bois, 1957), as well as against authoritarian regimes worldwide (Jahanbegloo, 2021). Moreover, it continues to be actively stimulating in today’s struggles for democratization (Mohanty, 2021), for example, opposing exclusionary populism (Gianolla, 2020; Rai and Tiwary, 2021) and supporting environmental justice (Shiva, 2021). Gandhi went beyond progressive political cultures to reflect on social love with his deconstruction of possessive individualism and the reconstruction of a non-individualistic and non-possessive community. This was an audacious attempt to undermine the hegemony of Western colonial politics that is an unmatched achievement compared to contemporary political thought. The analysis above shows that Gandhi’s political culture uncovers problematic aspects of liberal democracy with a nuanced understanding of the moral, cognitive, and emotional issues that limit liberal and critical political cultures. Duty in Gandhi’s thought generates democratic agency, subjectivity, and relationality; it expands equality and deepens the recognition of humanity, therefore it makes (human) rights more consistent by strengthening the political, moral, and material conditions for their effective realization. Honneth (1995) demonstrates how self and social realization derives from recognition at three levels: intimate love generates self-confidence, legal recognition generates self-respect, and socio-cultural solidarity generates self-realization. This model outlines a process in which “‘lack of coercion’ and ‘freedom’ cannot be understood simply as the absence of external force or influence, but must rather signify the lack of inner barriers as well as psychological inhibitions and fears” (Honneth, 1995, p. 174). However, Honneth is unable to see how social love can be constructed beyond in-group solidarity, which is associated with achieving common goals (Iorio and Campello, 2013). Therefore, contrasting with what is assumed by Cataldi and Iorio (2022b, p, 14) for social love where inter-groups differentiation prevails, recognition in *Satyagraha* exceeds the in-group solidarity outlined by Honneth. Gandhi’s constructivist postcolonial political sociology points out individual and collective pathways to configure social struggle and organization on relations of love. Social love in Gandhi’s *Satyagraha* is not an obligation but a liberating duty, a duty that realizes the self in the relation with the other beyond one’s in-group.

Gandhi has elevated the attention to social love beyond the private or in-group sphere; his focus on *swaraj* as self-rule implies both the individual and social re-joining in pursuing love in the struggle for power. This approach is aligned with the dismantling of the private–public divide, as famously elaborated by feminist scholarship, and produces a fine reassessment of recognition (Connolly, 2010). Firmness and courage are the emotional premises for non-violence, self-rule, relationality, and democracy framed as social love. This contrasts with the exclusionary populist politics of fear, grievence, and anxiety that struggle to dominate around the world by portraying the necessity of inter-group aversion and social distrust.

Gandhi advocated social love to the extent that colonizers and colonized could mutually support each other (Parel, 2003). Likewise, he did it with high caste Hindus, Dalits, and Muslims within Indian civilization. Gandhi’s inter-civilizational approach was original and a mediation of Eastern and Western philosophies (Bandopadhyay, 2021). This approach overcame cultural and race-based exclusionary practices, instead welcoming dialogue, which ultimately benefits both the emancipated and the colonizer reconciled with “true” equilibrated life. This approach tackled the very idea of colonialism and capitalism by criticizing the centrality of wealth and power over morality and spirituality. Anti-colonialism was a constitutive component of social love in Gandhi, and its achievement is related to engaged social dialogue.

Gandhi advanced experienced humanness above abstract ideals; action for him was thus much more related to real human life than with general ideal principles (Mehta, 2022). Compared to abstract morality, Gandhi’s political culture of *Satyagraha* leads to constructivist spiritual empiricism, the formation of the subject pursued and expanded through experimentalism. “Gandhi was not a system builder. He was essentially a pathfinder toward social and individual goals […] in order to go forward in the path of liberation from selfishness and injustice to selflessness and welfare of all” (Jahanbegloo, 2018, p. 97). This article thus shows the fine and open-to-change characteristics of Gandhi’s *Satyagraha*, a political culture of emancipation, horizontality, and diversity operated by social love.
